# Effects of egg yolk choline intake on cognitive functions and plasma choline levels in healthy middle-aged and older Japanese: a randomized double-blinded placebo-controlled parallel-group study

**DOI:** 10.1186/s12944-023-01844-w

**Published:** 2023-06-20

**Authors:** Soyogu Yamashita, Naoki Kawada, Wei Wang, Kenta Susaki, Yumi Takeda, Mamoru Kimura, Yoshitaka Iwama, Yutaka Miura, Michihiro Sugano, Ryosuke Matsuoka

**Affiliations:** 1 R&D Division, Kewpie Corporation, 2-5-7, Sengawa-Cho, Chofu-Shi, Tokyo, 182-0002 Japan; 2 Nihonbashi Cardiology Clinic, 13-4, Nihonbashi-Kodenmacho, Chuo-Ku, Tokyo, 103-0001 Japan; 3 Tokyo University of Agriculture and Technology, 3-8-1, Harumi-Cho, Fuchu-Shi, Tokyo, 183-8538 Japan; 4 Professor emeritus of Kyushu University and Prefectural University of Kumamoto, Kyusyu, Japan

**Keywords:** Phosphatidylcholine, Egg yolk, Cognitive function, Verbal memory, Clinical trial

## Abstract

**Background:**

Choline, as a neurotransmitter acetylcholine precursor, is reportedly associated with cognitive function. Although there are several cohort and animal studies on choline-containing foods and cognitive function, only a few interventional studies were reported. Egg yolk is a rich source of different choline-containing chemical forms, such as phosphatidylcholine (PC), lysophosphatidylcholine (LPC), and α-glycerophosphocholine (α-GPC). This study aimed to investigate the effect of consuming 300 mg of egg yolk choline per day on cognitive function of Japanese adults.

**Methods:**

A 12-week, randomized, double-blind, placebo-controlled, parallel-group study was conducted in 41 middle-aged and elderly males and females (43.9% female) aged ≥ 60 years and ≤ 80 years without dementia. Participants were randomly assigned to placebo and choline groups. The choline group received a supplement containing egg yolk choline (300 mg/day), and the placebo group received an egg yolk supplement free from choline for 12 weeks. Assessments of Cognitrax, Trail Making Tests (TMT) part A and B, the MOS 36-Item Short-Form Health Survey (SF-36), the Simplified Japanese Version of the WHO-Five Well-Being Index (WHO-5), and plasma choline levels were performed before and 6 and 12 weeks after supplement intake. In the present study, 19 subjects (9 in the placebo group and 10 in the choline group) were excluded due to the violation of the discontinuation criteria or participant compliance, and 41 subjects were analyzed.

**Results:**

The change amount of verbal memory scores and verbal memory test-correct hit (delay) was significantly higher in the choline group than in the placebo group at baseline-6 and baseline-12 weeks. The plasma free choline level was significantly higher in the choline group compared with the placebo group at 6 weeks. Conversely, the choline group showed significantly lower Cognitrax processing speed scores, symbol digit coding testing correct responses, and SF-36 physical quality of life summary scores compared to the placebo group at 6 weeks.

**Conclusions:**

The results suggested that continued 300 mg/day intake of egg yolk choline improved verbal memory, which is a part of cognitive functions. To confirm the observed effects of egg yolk choline, more well-designed and large-scale studies are warranted.

**Trial registration:**

Study protocols were pre-registered in the Clinical Trials Registration System (UMIN-CTR) (UMIN 000045050).

## Background

Recently, population aging has progressed worldwide. The number of people aged ≥ 60 years is expected to increase by 2 billion till 2050 [1]. Accompanying with this dramatic increase in aged population, the number of people affected by dementia is also increasing currently, with an estimated 132 million in 2050 globally[2]. Conditions that are not dementia but have mild cognitive decline are referred to as mild cognitive impairment (MCI). People with MCI are at risk for conversion to dementia while it has been known to recover to normal cognitive function. Since recovering to normal cognitive function is impossible once the cognitive status of dementia is achieved, it is important to prevent dementia at the stage of MCI before dementia [3–6].

Exercise habituation, smoking cessation, nutritional intervention, and social activity are recommended to reduce the risk of developing dementia [7]. In a study conducted in Japan, one of the foods that are expected to be effective in preventing dementia is chicken eggs, which is listed as one of the foods recommended to increase its consumption in old age [8]. A prospective cohort study in Finland reported that individuals with high egg ingestion have better cognitive function than those with low egg intake [9], suggesting association with egg intake and cognitive function.

PC, one of the choline compound in the egg yolk, is the popular source of choline, a precursor to the neurotransmitter acetylcholine, which is hypothesized to help maintain cognitive function [10, 11]. The amount of acetylcholine in the brain decreases with age due to a lack of the enzyme that converts choline to acetylcholine, resulting in decline of memory. Acetylcholine decrease also causes Alzheimer’s disease, a type of dementia [12]. In animal studies, significant differences were reported in memory and learning abilities between mice fed a free choline-deficient diet and a free choline-enriched diet [13]. Furthermore, administration of PC from hen’s eggs increased brain acetylcholine levels and improved memory and learning abilities [14, 15]. In contrast, intake of lecithin after the progression of Alzheimer’s dementia showed no improved effect on cognitive function, hypothesizing the importance of taking a choline compound before cognitive decline progresses [16].

The choline compounds in egg yolk are composed primarily PC, and its metabolites, LPC and α-GPC . They are collectively called egg yolk choline. Although there are various chemical forms of choline, we focused on egg yolk choline because of the high proportion of PC, the phospholipid form in foods.

Therefore, a 12-week, randomized, double-blind, placebo-controlled, parallel-group study was conducted to investigate the effect of continuous intake of egg yolk choline on cognitive function. Subjects were healthy, middle-aged, and elderly Japanese males and females aged 60 to 80 years without dementia who were concerned or had been pointed out by others about their forgetfulness.

## Methods

### Subjects

The participants were 60 middle-aged and elderly Japanese males and females aged 60–80 years without dementia who had been made aware of their forgetfulness or had it pointed out by others. Moreover, they were judged not to be ill by a clinical investigator and with 26 points or more on the Mini-Mental State Examination-Japanese (MMSE-J) screening test [17, 18]. Exclusion criteria included the following: a history of psychiatric disorders, cerebrovascular diseases, or other serious diseases; a Geriatric Depression Scale Short Version-Japanese (GDS-S-J) of ≥6 points in the screening test [19]; use of medicines that may affect the central nervous system, foods with health claims to improve cognitive function, and other medicines that may affect the test results; regular use of foods with health claims to improve blood flow or have an antioxidative effect, which may contribute to improving cognitive function; impaired in vision and hearing; impaired daily living activities; excessive smoking and alcohol consumption; extremely irregular dietary habits; allergies to the supplement ingredients; participation in other studies; and those who were judged by the investigator to be inappropriate as participants.

The sample size was determined according to a previous pilot study [20]. The number of samples required was ≥ 39 when type 1 risk α was 0.05 and power 1-β was 80%. The number of participants in this study was 60, 30 per group, to ensure this number of people. Sample size calculations were according to Cancer research and biostatistics statistical tools (https://stattools.crab.org/). The investigator fully explained the purpose and content of the study to the participants and provided written consent based on the participants free will before conducting the study.

### Study design

The study was conducted at KSO Inc. (Tokyo, Japan) from August to December 2021, under the supervision of Iwama Y. as the principal investigator with support from a project team composed of a doctor, a clinical laboratory technician, and a nutritionist. This 12-week, randomized, double-blind, placebo-controlled, parallel-group study divided the participants into the placebo and choline groups by the block-randomization method.

The Cognitrax test [21] and Trail Making Tests (TMT) part A and B [22] were performed for the assessment of cognitive function. To assess the quality of life (QOL) of participants, MOS 36-Item Short-Form Health Survey (SF-36) [23, 24] and the Simplified Japanese Version of the WHO-Five Well-Being Index (WHO-5) [25] were performed before and 6 and 12 weeks after intake of capsules. The cognitive function test was performed in a private room with one clinical psychologist per participant and no other interference. Additionally, blood was withdrawn to analyze the plasma choline level at each time point. Dietary surveys with brief-type self-administered diet history questionnaires (BDHQs) were performed [26, 27] and blood and urine samples were taken for safety evaluation before and 12 weeks after intake.

**Fig. 1:**
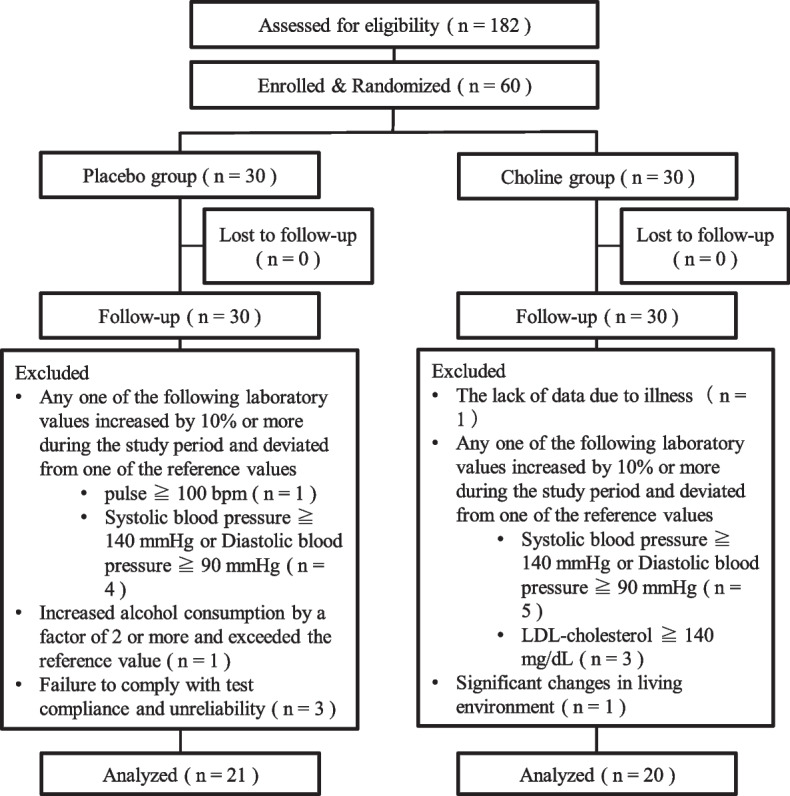
Flow chart

There were no dropouts during the study period, but five participants were excluded due to a substantial change in the living environment during the study period, a violation of study limitations, and a lack of reliability. One participant could not take the 12-weeks test on that day due to illness, and only a safety evaluation was conducted six days later. Furthermore, 14 subjects were excluded because they had 10% or more changes in the clinical test that have been reported to be associated with cognitive function [28, 29], before and after intake and deviated from one or more of the criteria (systolic blood pressure of < 140 mmHg, diastolic blood pressure of < 90 mmHg, pulse rate of < 100 bpm, and LDL-cholesterol of < 140 mg/dL). Thus, the analysis included 41 participants with 20 in the placebo group and 21 in the choline group. The detailed flow diagram of the study is shown in Figure 1.

### Supplements

The supplements used in this study were soft capsule-shaped and were manufactured by Aliment Industry Co., Ltd. (Yamanashi, Japan). In the previous study, it was reported that 300 mg of egg yolk choline per day maintained verbal memory, one of the typical cognitive functions [20]. Therefore, egg yolk oil, including 300 mg of egg yolk choline, was included (Kewpie Corporation, Tokyo, Japan) in supplements for the choline group, and lecithin-free egg yolk oil was included (Kewpie Corporation, Tokyo, Japan) in supplements for the placebo group. Table 1 shows the nutrient composition of each supplement. Participants consumed seven capsules per day after breakfast. Egg yolk choline is presented in PC equivalent and in theoretical value. The choline value is calculated from egg yolk choline.

**Table 1:** Nutrient composition of each capsule (/7 capsule)

		Placebo	Choline
Energy	(kcal)	21	22
Protein	(g)	0.7	0.7
Fat	(g)	1.5	1.5
Carbohydrates	(g)	0.4	0.4
Salt equivalent	(g)	0.0	0.0
Egg yolk choline in PC equivalent^*^	(mg)	0.0	289.6
Choline^†^	(mg)	0.0	38.8

^*^The value was presented as a theoretical value

^†^The value presented was calculated from egg yolk choline

### Primary endpoint

As the primary endpoint, the Cognitrax test (Health Solution Inc., Tokyo, Japan), a computer-based cognitive test with a Japanese version developed by CNS Vital Signs, LLC. (USA) [21], was administered. Cognitrax comprised 7 test items (verbal memory [VBM], visual memory [VIM], finger tapping [FTT], symbol digit coding [SDC], Stroop test [ST], shifting attention [SAT], and continuous performance [CPT]), and the results were shown as 11 domain scores (composite memory, VBM, VIM, psychomotor speed, reaction time, complex attention, cognitive flexibility, processing speed, executive function, simple attention, and motor speed).

### Secondary endpoints

As secondary endpoints, five tests were conducted. All tests other than safety evaluations were conducted before the test and 6 and 12 weeks after intake. Safety evaluations were conducted before the test and 12 weeks after intake.

#### Trail making test

TMT part A (TMT-A) and part B (TMT-B) were performed to assess attention and executive function. Participants were asked to connect the numbers from 1 to 25 in TMT-A and to connect the numbers from 1 to 13 and the 12 Japanese letters alternatively in TMT-B as early as possible. The time taken to perform the test and the number of errors were assessed.

#### SF-36

SF-36 consisted of 36 questions to measure eight health concept subscales (physical functioning, physical role, bodily pain, general health, vitality, social functioning, emotional role, and mental health). Additionally, physical component summary scores (PCS), mental component summary scores, and role/social component summary scores were calculated from eight subscales.

#### WHO-5

WHO-5 consisted of five questions (cheerful and in good spirits, calm and relaxed, active and vigorous, wake up feeling fresh and rested, things that interest me) to assess the condition in the last 2 weeks, and each score and total score were calculated.

#### Plasma choline level

Plasma free and fat-soluble choline levels were analyzed, respectively. 100 μL of plasma was mixed with 900 μL of methanol. Following sonication for 10 minutes, the samples were centrifuged at 15,000 rpm for 10 min. at 4°C, and the supernatant was aliquoted. Filter filtration was performed immediately before the analysis, and plasma free choline levels was measured by LC/MS/MS quantitative analysis (Waters, QTRAP4500, 40 °C, A: acetonitrile /B:5 mM ammonium acetate [pH 4.0]), and fat-soluble choline levels were also measured by LC/MS/MS quantitative analysis (BRUKER, micrOTOF-QII, 30°C, A: acetonitrile /B:5 mM ammonium acetate [pH4.0]). The analyses were conducted by the specialist in charge of the analyses of Kewpie Corporation (Tokyo, Japan).

#### Dietary survey with BDHQs

This was performed to ensure that subjects were not overeating or undereating, which would negatively influence the evaluation of the effects of functional foods.

#### Safety evaluation

As a safety evaluation, it was performed that, physical measurement (height, weight, and body mass index), physical examination (systolic blood pressure, diastolic blood pressure, and pulse), hematological tests (leukocyte count, erythrocyte count, hematocrit, and platelet count), blood biochemistry tests (total protein, albumin, total bilirubin, indirect bilirubin, alkaline phosphatase, aspartate transaminase, alanine transaminase, L-lactate dehydrogenase, gamma-glutamyl transpeptidase, total cholesterol, triglycerol, high-density lipoprotein-cholesterol, urea-nitrogen, creatinine, sodium, potassium, chloride, blood glucose, and hemoglobin A1C), urinalysis (protein, glucose, and occult blood reaction), and medical interviews. Blood and urine analyses were conducted at BML, Inc. (Saitama, Japan). Blood collection was performed after fasting for 4 h or more. The participants completed daily life diaries to investigate the status of consumption of the supplements and the presence of adverse events.

### Ethical approval

The study was conducted after obtaining approval from the Nihonbashi Cardiology Clinic Institutional Review Board (approval number: NJI-021-07-01, approval date: July 27, 2021), in accordance with the principles of the Declaration of Helsinki by the World Medical Association and the ethical guidelines for bioscience and medical research involving human subjects set forth by Ministry of Education, Culture, Sports, Science and Technology, Ministry of Health, Labour and Welfare, and Ministry of Economic, Trade, and Industry. The study protocol was pre-registered with the Clinical Trials Registry System (UMIN-CTR) (UMIN 000045050).

### Statistical analysis

Allocation-adjustment factors were age, sex, egg consumption, plasma free choline level, Cognitrax short version total and verbal memory scores, MMSE-J score, and GDS-S-J score. The reason why the verbal memory score was added to the adjustment factor is that the function was confirmed by previous research [20]. The allocation was conducted by the head of statistical analysis, who was not involved in laboratory tests.

The test results are presented as the mean ± standard error of the amount of change. The statistical significance level was set at *P* <0.05. Statistical analysis was performed using the computer software IBM Statistical Package for the Social Sciences version 28.0 (IBM Japan Co., Ltd. (Tokyo, Japan)). The change amounts of Cognitrax, TMT-A and B, and plasma choline levels were compared between the choline and placebo groups at different time points by a Student’s* t*-test. Mann-Whitney’s U test was performed to compare SF-36 and WHO-5 between the choline and placebo groups at different time points. To evaluate safety assessments, paired *t*-test was performed within groups, and student* t*-test was performed between groups.

## Results

### Subject characteristics

Participant characteristics are shown in Table 2. No significant differences were found between the two groups. Additionally, the sample consumption rate was 99%.

**Table 2:** Participant characteristics

		Placebo			Choline			*P*-value
		Mean	±	SE	Mean	±	SE	
Number of subjects		21 (11: 10)			20 (12: 8)			
(Male: Female)								
Age (y)		66	±	1.22	64.7	±	0.67	> 0.05
Height (cm)		160	±	1.72	163	±	1.62	> 0.05
Body weight (kg)		60.1	±	2.29	60.4	±	2.64	> 0.05
BMI		23.4	±	0.75	22.3	±	0.65	> 0.05
Systolic blood pressure (mmHg)		121	±	3.67	125	±	3.47	> 0.05
Diastolic blood pressure (mmHg)		76.7	±	2.67	76.8	±	2.81	> 0.05
Pulse (/m)		79.7	±	2.46	78.1	±	2.04	> 0.05
MMSE-J		28.8	±	0.21	28.9	±	0.24	> 0.05
GDS-S-J		1.33	±	0.3	1.7	±	0.35	> 0.05
Cognitrax	Verbal memory	52.7	±	1.11	50.9	±	1.42	> 0.05
	Psychomotor speed	162	±	3.66	160	±	5.7	> 0.05
	Processing Speed	53	±	1.62	55.8	±	1.83	> 0.05
	Simple Attention	39.8	±	0.11	39.4	±	0.4	> 0.05
	Motor Speed	108	±	2.84	104	±	4.98	> 0.05
Plasma free choline level (μM)		14.3	±	0.59	14.3	±	0.67	> 0.05
Egg consumption (n/week)		2.84	±	0.44	3.39	±	0.39	> 0.05

Mean ± SE of 20 or 21 subjects. The *P*-values are from the Student’s t-test, comparing values in the placebo and choline groups

### Cognitrax

The results of the cognitive function scores and the results of each test score are shown in Table 3 and Table 4, respectively. At 6 and 12 weeks after intake, the verbal memory score and the number of correct hits (delayed) on verbal memory tests were significantly higher in the choline group than in the placebo group. Conversely, at 6 weeks after intake, the processing rate score and correct response on the SDC test were significantly lower in the choline group than in the placebo group.

**Table 3:** Effects of egg yolk choline on ⊿ Cognitrax Score in subjects

	group	n	⊿ 6 weeks				⊿ 12 weeks			
			Mean	±	SE	*P*-value	Mean	±	SE	*P*-value
Composite Memory	Placebo	21	− 0.90	±	1.59	0.569	− 0.38	±	1.27	0.470
	Choline	20	0.40	±	1.61		1.10	±	1.59	
Verbal Memory	Placebo	21	− 0.33	±	0.57	0.003^**^	0.33	±	0.73	0.043^*^
	Choline	20	3.35	±	1.00		2.75	±	0.90	
Visual Memory	Placebo	21	− 0.57	±	1.30	0.213	− 0.71	±	1.07	0.550
	Choline	20	− 2.95	±	1.36		− 1.65	±	1.13	
Psychomotor speed	Placebo	21	3.81	±	1.69	0.711	4.43	±	1.95	0.834
	Choline	20	2.75	±	2.31		3.80	±	2.27	
Reaction Time	Placebo	21	2.76	±	10.7	0.941	0.62	±	10.7	0.418
	Choline	20	1.30	±	16.6		− 13.15	±	13.1	
Complex Attention	Placebo	21	− 0.81	±	0.68	0.894	− 1.14	±	0.84	0.995
	Choline	20	− 0.95	±	0.81		− 1.15	±	0.67	
Cognitive Flexibility	Placebo	21	2.14	±	1.47	0.317	3.76	±	1.92	0.458
	Choline	20	4.30	±	1.54		5.60	±	1.51	
Processing Speed	Placebo	21	2.86	±	1.53	0.045^*^	2.43	±	1.60	0.414
	Choline	20	− 1.40	±	1.37		0.85	±	1.01	
Exclusive Function	Placebo	21	1.57	±	1.58	0.175	3.57	±	1.82	0.325
	Choline	20	4.60	±	1.51		5.95	±	1.53	
Simple Attention	Placebo	21	− 0.10	±	0.14	0.296	0.00	±	0.14	0.684
	Choline	20	0.20	±	0.25		0.10	±	0.20	
Motor Speed	Placebo	21	0.90	±	1.32	0.178	2.24	±	1.21	0.734
	Choline	20	3.95	±	1.81		3.05	±	2.07	

Mean ± SE of 20 or 21 subjects. The *P*-values are from the Student’s t-test, comparing values between the placebo and choline groups

^*^P < 0.05

^**^P < 0.01

**Table 4:** Effects of egg yolk choline on ⊿ Cognitrax test score in subjects

		group	n	⊿ 6 weeks				⊿ 12 weeks			
				Mean	±	SE	*P*-value	Mean	±	SE	*P*-value
Verbal Memory Test (VBM)	Correct Hits (immediate)	Placebo	21	− 0.14	±	0.43	0.213	0.24	±	0.37	0.736
		Choline	20	0.55	±	0.34		0.40	±	0.30	
	Correct Passes (immediate)	Placebo	21	− 0.05	±	0.16	0.221	0.00	±	0.17	0.241
		Choline	20	0.30	±	0.23		0.35	±	0.24	
	Correct Hits (delay)	Placebo	21	− 0.10	±	0.37	0.015^*^	− 0.24	±	0.54	0.046^*^
		Choline	20	1.95	±	0.72		1.60	±	0.72	
	Correct Passes (delay)	Placebo	21	− 0.05	±	0.26	0.131	0.33	±	0.16	0.801
		Choline	20	0.55	±	0.29		0.40	±	0.21	
Visual Memory Test (VIM)	Correct Hits (immediate)	Placebo	21	− 0.48	±	0.55	0.707	− 0.10	±	0.44	0.22
		Choline	20	− 0.75	±	0.46		− 0.85	±	0.42	
	Correct Passes (immediate)	Placebo	21	− 0.24	±	0.62	0.423	0.14	±	0.49	0.478
		Choline	20	− 0.85	±	0.42		− 0.35	±	0.48	
	Correct Hits (delay)	Placebo	21	0.00	±	0.48	0.349	− 0.43	±	0.45	0.803
		Choline	20	− 0.75	±	0.64		− 0.25	±	0.56	
	Correct Passes (delay)	Placebo	21	0.14	±	0.49	0.378	− 0.33	±	0.58	0.868
		Choline	20	− 0.60	±	0.68		− 0.20	±	0.55	
Finger Tapping Test (FTT)	Right Tap Average	Placebo	21	0.90	±	1.04	0.275	1.76	±	0.87	0.838
		Choline	20	2.55	±	1.06		2.05	±	1.11	
	Left Tap Average	Placebo	21	0.00	±	0.48	0.144	0.48	±	0.54	0.664
		Choline	20	1.40	±	0.82		1.00	±	1.06	
Symbol Digit Coding Test (SDC)	Correct Responses	Placebo	21	2.90	±	1.21	0.021^*^	2.19	±	1.33	0.382
		Choline	20	− 1.20	±	1.19		0.75	±	0.91	
	Errors	Placebo	21	0.05	±	0.38	0.762	− 0.24	±	0.38	0.772
		Choline	20	0.20	±	0.32		− 0.10	±	0.27	
Stroop Test (ST)	Simple reaction time	Placebo	21	− 0.95	±	8.76	0.549	− 5.81	±	6.10	0.880
		Choline	20	− 16.0	±	23.15		− 9.40	±	22.74	
	Complex Reaction Time	Placebo	21	0.38	±	11.46	0.606	− 1.67	±	13.46	0.481
		Choline	20	12.1	±	19.3		− 14.6	±	12.2	
	Stroop Reaction Time	Placebo	21	5.38	±	14.51	0.509	3.14	±	13.18	0.524
		Choline	20	− 9.80	±	17.66		− 11.55	±	18.93	
	Stroop Commission Errors	Placebo	21	− 0.57	±	0.36	0.058	− 0.19	±	0.26	0.103
		Choline	20	0.30	±	0.25		0.35	±	0.20	
Shifting Attention Test (SAT)	Correct Responses	Placebo	21	1.24	±	0.94	0.104	2.62	±	1.15	0.222
		Choline	20	3.55	±	1.03		4.55	±	1.05	
	Errors	Placebo	21	− 0.33	±	0.74	0.465	− 0.95	±	0.75	0.645
		Choline	20	− 1.05	±	0.62		− 1.40	±	0.60	
	Correct Responses Time	Placebo	21	− 26.67	±	21.16	0.323	− 40.19	±	23.1	0.123
		Choline	20	− 59.65	±	25.44		− 90.90	±	22.28	
Continuous Performance Test (CPT)	Correct Responses	Placebo	21	− 0.10	±	0.07	0.259	− 0.05	±	0.05	0.427
		Choline	20	0.10	±	0.16		0.05	±	0.11	
	Commission Errors	Placebo	21	0.10	±	0.07	0.259	0.05	±	0.05	0.427
		Choline	20	− 0.10	±	0.16		− 0.05	±	0.11	
	Errors	Placebo	21	0.00	±	0.10	0.527	− 0.05	±	0.13	0.990
		Choline	20	− 0.10	±	0.12		− 0.05	±	0.14	
	choice reaction time	Placebo	21	8.19	±	5.34	0.857	− 2.57	±	5.25	0.675
		Choline	20	6.60	±	7.00		1.60	±	8.48	

Mean ± SE of 20 or 21 subjects

^*^statistical significance by Student’s t-test for between-group at *P* < 0.05

### TMT

The results of the TMT scores are shown in Table 5. No significant differences could be identified at any time point during the study period.

**Table 5:** Effects of egg yolk choline on ⊿TMT test scores in subjects

		group	n	⊿ 6 weeks				⊿ 12 weeks			
				Mean	±	SE	*P*-value	Mean	±	SE	*P*-value
TMT-A	Time (sec)	Placebo	21	− 5.24	±	1.48	0.183	− 4.67	±	1.68	0.415
		Choline	20	− 2.20	±	1.7		− 6.80	±	1.99	
	Error	Placebo	21	− 0.14	±	0.1	0.106	− 0.10	±	0.1	0.681
		Choline	20	0.05	±	0.05		− 0.05	±	0.05	
TMT-B	Time (sec)	Placebo	21	− 15.67	±	6.63	0.070	− 18.62	±	6.64	0.134
		Choline	20	− 0.60	±	4.49		− 6.95	±	3.55	
	Error	Placebo	21	− 0.19	±	0.23	0.474	− 0.05	±	0.28	0.871
		Choline	20	0.05	±	0.25		− 0.10	±	0.14	

Mean ± SE of 20 or 21 subjects. The *P*-values are from the Student’s t-test, comparing values between the placebo and choline groups

### Plasma choline concentration

The results of plasma choline levels are shown in Table 6. Free choline levels were significantly higher in the choline group compared with the placebo group at 6 weeks after ingestion. The choline group remained at a high level after 12 weeks of ingestion, though not significant. No significant changes were observed in fat-soluble choline concentrations between groups.

**Table 6:** Effects of egg yolk choline on plasma choline levels in subjects

	group	n	⊿ 6 weeks				⊿ 12 weeks			
			Mean	±	SE	*P*-value	Mean	±	SE	*P*-value
Plasma free choline level (μM)	Placebo	21	0.34	±	0.51	0.039^*^	0.04	±	0.58	0.415
	Choline	20	1.80	±	0.46		1.06	±	0.65	
Plasma fat-soluble choline level (μM)	Placebo	21	− 0.14	±	0.10	0.106	87.9	±	68.8	0.681
	Choline	20	0.05	±	0.05		117.5	±	42.1	

Mean ± SE of 20 or 21 subjects

^*^statistical significance by Student’s t-test for between-group at *P* < 0.05

### QOL survey

SF-36 revealed significantly lower PCS 12 weeks post-ingestion in the choline group compared with the placebo group (1.88 ± 1.1vs. −1.76 ± 1.19; *P* = 0.029). No significant differences were found in WHO-5 among all parameters (data not shown).

### Dietary survey with BDHQs

The results of the dietary survey with BDHQs are shown in Table 7. At 0 week, the placebo group consumed significantly less protein than the choline group. At 12 weeks, the fat intake in the placebo group was significantly lower than at 0 week.

**Table 7:** Dietary survey with BDHQs

	group	n	0 week				12 weeks				*P*-value2
			Mean	±	SE	*P*-value1	Mean	±	SE	*P*-value1	
Energy	Placebo	21	1864	±	143	0.122	1664	±	130	0.426	0.090
	Choline	20	1571	±	117		1526	±	110		0.585
Protein	Placebo	21	74.9	±	7.2	0.048^*^	68.1	±	6.9	0.276	0.095
	Choline	20	58.0	±	3.9		59.1	±	4.0		0.737
Fat	Placebo	21	60.3	±	5.2	0.152	52.2	±	4.6	0.642	0.004^†^
	Choline	20	50.6	±	4.0		49.3	±	4.0		0.637
Carbohydrates	Placebo	21	233	±	15.3	0.280	215	±	15.2	0.402	0.370
	Choline	20	206	±	18.7		196	±	15.9		0.518
Salt equivalent	Placebo	21	11.5	±	1.1	0.068	10.5	±	0.9	0.436	0.111
	Choline	20	9.3	±	0.5		9.7	±	0.6		0.448

Mean ± SE of 20 or 21 subjects

^*^statistical significance by Student’s t-test for between-group at *P* < 0.05

^†^statistical significance by Paired t-test for within-group at *P* < 0.05

### Safety evaluation

The results of the blood and urine analyses are shown in Table 8. Any changes were within the normal range, although significant changes were observed in some parameters. In physical measurement, there were no significant changes (Date not shown). The investigator judged that continuous egg yolk choline ingestion at 300 mg for 12 weeks had no safety problems.

**Table 8:** Blood and urine analyses parameter

		Group	0 week					12-week			
			n	Mean	±	SE		n	Mean	±	SE
Hematological Tests	WBC (/μL)	Placebo	30	5197	±	195		30	5137	±	221
		Choline	30	5187	±	186		30	4917	±	185
	RBC (× 10^4^/μL)	Placebo	30	457	±	7		30	456	±	7
		Choline	30	458	±	8		30	464	±	8
	Hemoglobin (g/dL)	Placebo	30	14.2	±	0.2		30	14.3	±	0.2
		Choline	30	14.1	±	0.3		30	14.3	±	0.2
	Hematocrit (%)	Placebo	30	43.9	±	0.6		30	44.6	±	0.6
		Choline	30	43.6	±	0.7		30	45.0	±	0.7
	Platelet Count (× 10^4^/μL)	Placebo	30	23.2	±	1.0		30	23.6	±	0.8
		Choline	30	24.8	±	1.0		30	26.2	±	1.0
	MCV (fL)	Placebo	30	96.2	±	0.8		30	98.0	±	0.7
		Choline	30	95.4	±	0.7		30	97.2	±	0.8
	MCH (pg)	Placebo	30	31.1	±	0.3		30	31.4	±	0.3
		Choline	30	30.7	±	0.3		30	30.8	±	0.2
	MCHC (%)	Placebo	30	32.3	±	0.2		30	32.0	±	0.2
		Choline	30	32.2	±	0.1		30	31.7	±	0.1
	NEUT (%)	Placebo	30	54.9	±	1.4	^*^	30	56.3	±	1.5
		Choline	30	59.4	±	1.4		30	58.5	±	1.2
	LYMPH (%)	Placebo	30	36.2	±	1.4	^*^	30	34.8	±	1.3
		Choline	30	31.8	±	1.2		30	32.5	±	1.0
	MONO (%)	Placebo	30	5.61	±	0.14		30	5.76	±	0.19
		Choline	30	5.51	±	0.25		30	5.59	±	0.28
	EOSINO (%)	Placebo	30	2.55	±	0.33		30	2.43	±	0.38
		Choline	30	2.61	±	0.32		30	2.71	±	0.28
	BASO (%)	Placebo	30	0.770	±	0.078		30	0.730	±	0.059
		Choline	30	0.693	±	0.057		30	0.733	±	0.056
Blood Biochemistry Tests	AST(GOT) (U/L)	Placebo	30	22.1	±	0.81		30	25.1	±	1.51
		Choline	30	21.8	±	0.78		30	23.6	±	1.00
	ALT(GPT) (U/L)	Placebo	30	19.6	±	1.37		30	20.3	±	1.38
		Choline	30	17.6	±	1.33		30	18.3	±	1.48
	LD(LDH) (U/L)	Placebo	30	202	±	4		30	205	±	5
		Choline	30	193	±	4		30	204	±	5
	Total Bilirubin (mg/dL)	Placebo	30	0.913	±	0.052		30	0.877	±	0.044
		Choline	30	0.890	±	0.041		30	0.847	±	0.046
	ALP (U/L)	Placebo	30	69.7	±	3.1		30	71.0	±	3.1
		Choline	30	76.3	±	4.7		30	77.1	±	4.2
	γ-GT (γ-GTP) (U/L)	Placebo	30	27.8	±	2.9		30	26.6	±	2.5
		Choline	30	24.6	±	2.9		30	25.0	±	3.4
	Glucose (mg/dL)	Placebo	30	91.7	±	1.8		30	89.5	±	1.4
		Choline	30	89.0	±	1.7		30	89.8	±	1.9
	HbA1c (%)	Placebo	30	5.49	±	0.05		30	5.46	±	0.06
		Choline	30	5.51	±	0.04		30	5.48	±	0.05
	Total Cholesterol (mg/dL)	Placebo	30	225	±	6		30	228	±	6
		Choline	30	222	±	6		30	230	±	6
	LDL-Cholesterol (mg/dL)	Placebo	30	132	±	5		30	133	±	5
		Choline	30	130	±	6		30	133	±	5
	HDL-Cholesterol (mg/dL)	Placebo	30	71.9	±	4.1		30	78.5	±	4.3
		Choline	30	69.5	±	3.4		30	77.4	±	4.1
	Triglyceride (mg/dL)	Placebo	30	107	±	15		30	85.4	±	10.0
		Choline	30	104	±	12		30	102	±	14
	Phospholipid (mg/dL)	Placebo	30	242	±	6		30	243	±	6
		Choline	30	241	±	5		30	248	±	6
	Total Protein (g/dL)	Placebo	30	7.35	±	0.07		30	7.25	±	0.08
		Choline	30	7.23	±	0.07		30	7.22	±	0.07
	Albumin (g/dL)	Placebo	30	4.39	±	0.04		30	4.40	±	0.04
		Choline	30	4.35	±	0.05		30	4.39	±	0.04
	BUN (UN) (mg/dL)	Placebo	30	15.8	±	0.57		30	16.2	±	0.76
		Choline	30	15.1	±	0.70		30	16.1	±	0.65
	Creatinine (mg/dL)	Placebo	30	0.847	±	0.027		30	0.834	±	0.028
		Choline	30	0.791	±	0.025		30	0.781	±	0.026
	Uric Acid (mg/dL)	Placebo	30	5.48	±	0.21		30	5.42	±	0.20
		Choline	30	5.55	±	0.24		30	5.42	±	0.23
	Na (mEq/L)	Placebo	30	141	±	0		30	140	±	0
		Choline	30	141	±	0		30	141	±	0
	Cl (mEq/L)	Placebo	30	103	±	0		30	103	±	0
		Choline	30	104	±	0		30	103	±	0
	K (mEq/L)	Placebo	30	4.25	±	0.06		30	4.18	±	0.05
		Choline	30	4.21	±	0.05		30	4.27	±	0.05
	Ca (mg/dL)	Placebo	30	9.47	±	0.06		30	9.32	±	0.04
		Choline	30	9.48	±	0.05		30	9.40	±	0.05
Urinalysis	Acidity (pH)	Placebo	30	6.30	±	0.14		30	6.27	±	0.13
		Choline	30	6.10	±	0.12		30	6.15	±	0.14
	Specific Gravity	Placebo	30	1.01	±	0.00		30	1.02	±	0.00
		Choline	30	1.02	±	0.00		30	1.02	±	0.00

Mean ± SE of 30 subject

^*^statistical significance by Student’s t-test for between-group at *P* < 0.05

^†^statistical significance by Paired t-test for within-group at *P* < 0.05

## Discussion

This study evaluated the effects of continuous egg yolk choline ingestion at 300 mg on cognitive function in middle-aged and older adults who were aware of healthy forgetfulness but not dementia or who had been indicated by others for forgetfulness. The results showed that there was a significant improvement in the choline group compared with the placebo group in verbal memory ability, a part of the cognitive function.

In this test, plasma choline concentration was measured. Previous studies revealed that phosphatidylcholine ingestion increases plasma free choline and brain acetylcholine levels [14, 15]. Conversely, low plasma free choline levels are associated with low cognitive function, suggesting that plasma free choline levels and cognitive function are closely related [30]. In the present study, ingestion of egg yolk choline also increased plasma free choline levels, suggesting contribution to the improvement of verbal memory. Moreover, it was challenging to look at the other neurotransmitters due to the limited blood samples. Further future investigations will be required to uncover the potential mechanisms of action, for instance, plasma levels.

The ingested egg yolk choline is well absorbed in the small intestine by a complex mechanism. PC is degraded to LPC by pancreatic phospholipase A2 in the small intestine [31], and a portion of LPC is further degraded to α-GPC by lysophospholipase [32]. Most of the LPC is absorbed in the small intestine, resynthesized to PC and transported as chylomicrons and then derived to the liver via the lymphatics [33]. On the contrary, α-GPC is also absorbed in the small intestine and transported via portal vein to the liver [34]. Egg yolk choline transported to the liver is released into the blood as free choline and transported to the brain [34]. It has also been reported that part of orally ingested egg yolk choline is hydrolyzed to free choline in the small intestine [35]. Free choline is believed to be absorbed by sodium-dependent transporters and transferred to circulation [36]. Choline transported to the brain is thought to cross the blood–brain barrier and reach to brain cells via the choline transporter CHT1 or CHT2 [33]. After reaching the neurons, choline and acetyl-CoA are converted into the neurotransmitter acetylcholine by choline acetyltransferase (acetyl-CoA: choline O-acetyltransferase; ChAT, EC 2.3.1.6). Acetylcholine released extracellularly at nerve terminals is implicated in memory and learning functions in the brain by stimulating muscarinic and nicotinic acetylcholine receptors [37, 38]. In the present study, egg yolk choline-containing PC, LPC, and α-GPC were ingested, and plasma free choline levels were confirmed to be elevated, suggesting that egg yolk choline served as an acetylcholine precursor in the brain, resulting in increased acetylcholine levels and the maintenance and improvement of verbal memory abilities involving acetylcholine receptors. No change was found in plasma fat-soluble choline levels, which were thought to be strongly influenced by blood phospholipid variations. However, the pharmacokinetics of plasma free and fat-soluble choline remained to be clarified.

The processing speed score and SDC test used to calculate the rate of processing efficiency were significantly higher in the placebo group after 6 weeks, although these parameters have been reported to be a function related to cholinergic nerves [39]. However, since this effect disappeared after 12 weeks, the possibility suggests that temporary factors caused fluctuations. The PCS score of SF-36 was lower in the choline group than in the placebo group. In this context, it is possible that the result was affected by the reduction of the amount of physical activity, because this study was conducted in the spreading phase of the COVID-19 infection [40]. Regarding the dietary survey, blood analyses, and urine analyses, significant differences were shown in some parameters. But these mean scores were within the recommended daily intake or normal range. In addition, there were no adverse events attributed to the supplement intake during the study period.

Soybean is also a well-known source of phospholipids, but egg yolk phospholipids have a high level of PC of approximately 80% compared with an approximately 30% in soybean [41]. In addition, soybean phospholipids contain more phosphatidylethanolamine and phosphatidylinositol than egg yolk phospholipids. In this context, since ethanolamine, a breakdown product of phosphatidylethanolamine, inhibits the passage of choline through the blood–brain barrier [42], choline intake as egg yolk phospholipids would be more effective for cognitive function [43]. Moreover, since free choline is readily degraded to trimethylamine by intestinal bacteria in the upper small intestine before absorption compared with PC, administration of choline as phosphatidylcholine is recommended [44, 45].

## Strength and limitation

The appearance of significant differences in VBM score (at 6 weeks: *P* = 0.003; at 12 weeks: *P* = 0.043) and the number of correct hits (delayed) on VBM tests (at 6 weeks: *P* = 0.015; at 12 weeks: *P* = 0.046) were the strengths of this study. However, this study has some limitations. The first is that no significant differences were observed in cognitive function other than VBM. Secondly, subjects were Japanese old-age people who had a healthy diet from a global perspective. Similar observations have been reported in studies of non-Japanese populations [46], but the proportions of choline sources and egg yolk ingested, differs among populations of different the countries [47]. Neither is choline listed in the dietary reference intake information in Japan, nor is the data for choline given in the Japanese food tables. In addition, there is a single nucleotide polymorphism that modulates choline requirements, and the expression of this gene is known to vary by race [48]. However, the change in choline requirement due to this gene’s expression is related to organ dysfunction, and the effect on cognitive function has not been reported. Therefore, ingesting egg yolk choline was considered effective in maintaining cognitive function regardless of race. Finally, people with dementia or depression didn’t participate and the effects on dementia or depression remain unclear.

In the future, to assess the effects of choline intake more accurately, dietary choline intake will need to be investigated in Japan, or intervention tests will need to be conducted in the area where choline is listed in the dietary reference intake information. Its effect on different age groups or populations other than the Japanese are yet to be elucidated.

## Conclusions

This study showed that in middle-aged and older Japanese males and females, ingestion of 300 mg/day egg yolk choline increased plasma free choline levels and improved verbal memory, a part of cognitive function. It has been proposed that the consumption of egg yolk choline could potentially maintain cognitive function in individuals without dementia. However, it remains unclear that whether the intake of egg yolk choline could prevent or reduce the incidence of dementia. Nevertheless, it has been suggested that incorporating regular consumption of egg yolk choline into the diet is an effective strategy for maintaining brain function in adults who are free of dementia.

## Data Availability

The dataset supporting the conclusions of this article is included within the article.

## Abbreviations

MCI: Mild cognitive impairment
PC: Phosphatidylcholine
LPC: Lysophosphatidylcholine
MMSE-J: Mini-Mental State Examination-Japanese
GDS-S-J: Geriatric Depression Scale Short Version-Japanese
TMT: Trail Making Test
SF-36: 36-Item Short-Form Health Survey
WHO-5: WHO-Five well-being index
QOL: Quality of life
BDHQs: Brief-type self-administered diet history questionnaires
VBM: Verbal memory
PCS: Physical component summary scores
QOL: Quality of life
SDC: Symbol digit coding
